# Characterization of Insulin-like Peptide (ILP) and Its Potential Role in Ovarian Development of the Cuttlefish *Sepiella japonica*

**DOI:** 10.3390/cimb44060170

**Published:** 2022-05-27

**Authors:** Zhenming Lü, Chenghao Yao, Shijie Zhao, Yao Zhang, Li Gong, Bingjian Liu, Liqin Liu

**Affiliations:** 1 National Engineering Laboratory of Marine Germplasm Resources Exploration and Utilization, College of Marine Science and Technology, Zhejiang Ocean University, Zhoushan 316022, China; nblzmnb@zjou.edu.cn (Z.L.); s20070700069@zjou.edu.cn (C.Y.); s19070700038@zjou.edu.cn (Y.Z.); gongli@zjou.edu.cn (L.G.); liubingjian@zjou.edu.cn (B.L.); 2 National Engineering Research Center for Facilitated Marine Aquaculture, Zhejiang Ocean University, Zhoushan 316022, China; z19095136070@zjou.edu.cn

**Keywords:** cephalopod, reproduction, ovarian development, RNA interference

## Abstract

The insulin-like peptide (ILP) family is well known for regulating reproduction in invertebrates, while its role in mollusks remains largely unknown. In this study, we first isolated and characterized the ILP gene in the cuttlefish *Sepiella japonica*. The full-length *Sj*ILP cDNA obtained was 926 bp and encoded a precursor protein of 161 amino acids. The precursor protein consisted of a signal peptide, a B chain, a C-peptide, and an A chain. It possessed the typical features of ILP proteins, including two cleavage sites (KR) and eight conserved cysteines. To define the function of *Sj*ILP, the expression of *Sj*ILP in different tissues and ovarian development stages were analyzed using qRT-PCR. *SjILP* was mainly expressed in the ovary, and its gene expression correlated with ovarian development. Furthermore, silencing *SjILP* using RNA interference (RNAi) dramatically decreased the expression levels of four ovarian-development-related genes (vitellogenin1, vitellogenin2, cathepsin L1-like, and follistatin). These data suggest the critical role of *SjILP* in the regulation of ovarian development in *S. japonica*.

## 1. Introduction

Insulin-like peptides (ILPs), the most widely distributed peptide among invertebrate and invertebrate species, are essential regulatory factors in animal growth, development, metabolism, and reproduction [1]. In mollusks, since the first identification of insulin-like peptides (MIPs) in the pond snail *Lymnaea stagnalis* [2], a variable number of ILPs have been identified in different species, including gastropods [3], bivalves [4,5], and cephalopods. Unlike their vertebrate counterparts, the primary source of ILPs in mollusks is the nervous system [6]. Beyond that, ILPs are also produced in other tissues, such as the gill [7], intestinal epithelial [8], digestive gland [9], adductor muscle [10], siphon, and foot [11]. These peptides are stored in the secretory granules in response to cellular or molecular stimuli. Once released, the peptides can diffuse to target cells, where they combine with their receptors to regulate multiple processes [12].

**Figure 1:**
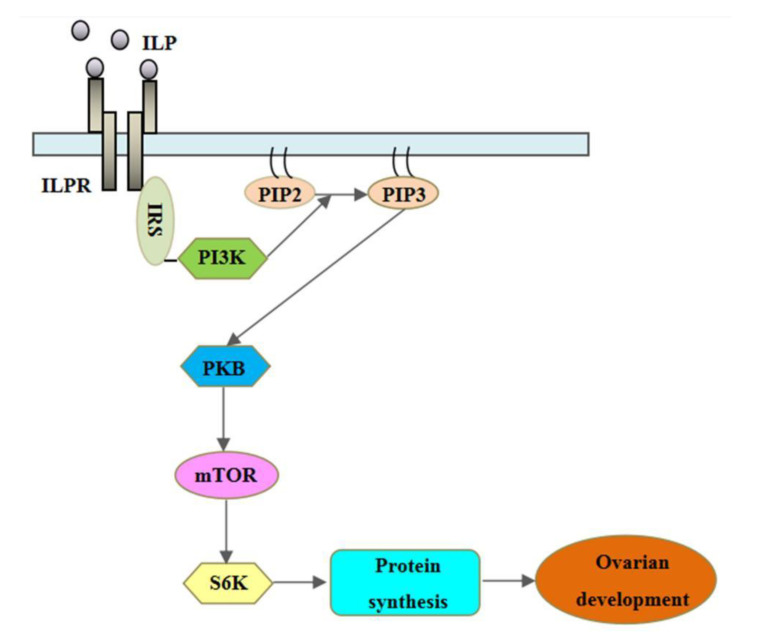
Proposed ILP signal transduction pathway in *S. japonica*. Abbreviations: IRS, insulin receptor substrate; PI3K, phosphatidylinositol-2,4,5-triphosphate; PIP2, phosphatidylinositol 4,5-bisphosphate; PIP3, phosphatidylinositol-3,4,5-triphosphate; PKB, protein kinase B; mTOR, mammalian target of rapamycin; S6K, ribosomal S6 protein kinase.

The biological functions of insulin observed in vertebrate species are generally conserved in invertebrates. In invertebrates such as mollusks, multiple ILPs are suggested to have functions other than just regulating animal growth [10], including the regulation of reproduction [13]. In *Planorbarius corneus* [14] and *Lymnaea stagnalis* [6], ILPs have been detected in the brain and are involved in the hormonal control of reproduction [15]. In isolated gonadal cells of *Helix aspersa*, Monnier and Bride [16] showed that vertebrate insulin stimulated protein synthesis, suggesting that ILPs may be involved in the regulation of germinal cell proliferation and maturation. In the Pacific oyster *Crassostrea gigas*, *Cg-*ILP is mainly expressed in the gonadal area, suggesting involvement in the control of sexual reproduction [5]. Combined with the finding that one receptor for *C. gigas* IRPs (CIR) is expressed in the gonadal area, these results suggest that ILPs are involved in reproduction via binding to the insulin receptor (IR) [13]. Genetic studies have indicated that multiple ILPs may activate this receptor and stimulate signal transduction through the PI3K/AKT pathway (Figure 1) [17].

The cuttlefish *Sepiella japonica* is one of the most important commercial species widely distributed in the Northwest Pacific Ocean and North Indian Ocean. However, due to the over-fishing of this species, the wild population has declined. Therefore, many artificial breeding programs have been established to increase the production of *S. japonica* [18]. However, it appeared to have sexual precocity in an artificial environment. Previous studies showed that the sexual precocity of female individuals was associated with ovarian development [19]. Furthermore, ovarian development is influenced by both genetic and environmental factors [20,21,22]. Among the genetic factors, some ovarian-development-related genes, such as *ILP*, have been indicated to be involved in this process in some molluscan species [13,14,17,23], while gene identification and functional analyses of it have not been conducted in cuttlefish. Only one ILP (QHI00148.1) in *Sepia latimanus* and two ILPs (ILP-7, XP_014782891.1, and ILP-3, XP_014772597.1) in *Octopus bimaculoides* have been identified, but their functions remain unclear. To the best of our knowledge, there is a paucity of relevant information regarding the presence of ILPs in cuttlefish. Moreover, these reports mainly concern the cloning and characterization of genes using RACE, expression profile using qRT-PCR, and cellular localization using situ hybridization. However, few studies have examined the functional characterization of ILPs in ovarian development via RNA interference.

Thus, considering the vital function of ILPs and the limited knowledge of cephalopods, this study aimed to investigate their potential involvement in ovarian development in *S. japonica*. In this study, we first identified the ILP gene from *S. japonica*. Then, we analyzed *Sj*ILP expression in different tissues and ovarian development stages using qRT-PCR. In addition, the expressions of several ovarian-development-related genes were determined after silencing *Sj*ILP. These data may provide valuable information for a better understanding of the molecular mechanisms of ovarian development in cephalopods.

## 2. Materials and Methods

### 2.1. Animals 

**Figure 2:**
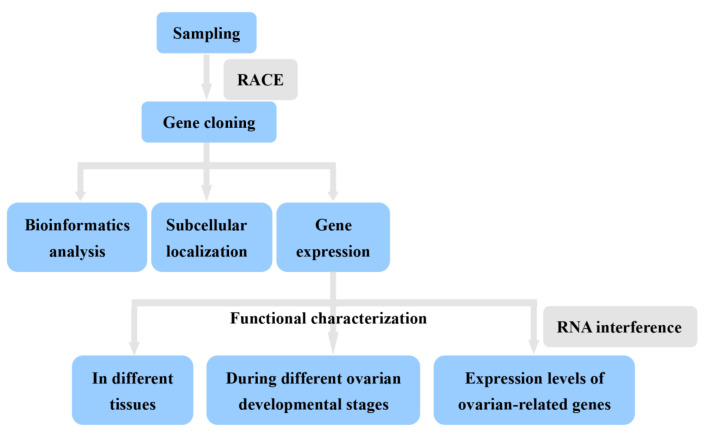
Roadmap of the experimental program.

Healthy adult *S. japonica* was collected from the aquaculture station of Marine Fisheries Research Institute of Zhejiang on Xishan island (Zhoushan, Zhejiang, China). Following a previous method [20], ovarian development was classified into the oogonium production period (stage I), protoplasmic growth period (stage II), interstitial growth period (stage III), and trophoplasmic growth period (stage IV) according to the color and size of the oocyte. To evaluate the expression of *Sj*ILP during the reproductive cycle of female *S. japonica*, animals in four ovarian development stages were collected. Before tissue sample collection, cuttlefish were anesthetized in a solution of magnesium chloride solution (27.0 g/L). Tissues were dissected, frozen immediately in liquid nitrogen, and then stored at −80 °C until RNA extraction. In Figure 2, we lay out a roadmap for this paper.

### 2.2. Cloning of SjILP

Total RNA was extracted from the ovary of adult *S. japonica* using Trizol reagent (Invitrogen, Carlsbad, CA, USA) following the manufacturer’s protocol. After RNA quality check using a NanoDrop 2000 spectrophotometer (Thermo Fisher Scientific, Waltham, MA, USA), 10µg of total RNA was reverse-transcribed into first-strand cDNA using M-MLV reverse transcriptase (RNase H^−^) (TaKaRa Bio Inc., Shiga, Japan).

A partial fragment of *ILP* was obtained from the transcriptome data of *S. japonica* [19]. Based on the partial fragment, specific primers were designed using the Primer 6.0 program (Table S4). The full-length cDNA of *SjILP* was determined using the rapid amplification of cDNA ends (RACE). PCR reaction was performed as described in Pang et al. [21]. The primers used in this study are shown in Table S1. PCR products were separated by electrophoresis on 1.5% agarose gel. Bands with the expected size were excised and cloned into the pGEM-Teasy vector (Promega) and then sequenced.

### 2.3. Bioinformatics Analysis 

The open reading frame (ORF) of ILP cDNA was predicted using the ORF Finder (https://www.ncbi.nlm.nih.gov/orffinder) (accessed on 10 February 2022) and checked using the BLAST program (https://blast.ncbi.nlm.nih.gov/Blast.cgi) (accessed on 10 February 2022) to confirm the identity of the sequence. The deduced amino acid sequence was subsequently used to predict the domain architecture using SMART (http://smart.embl.de/) (accessed on 10 February 2022). The signal peptide of the amino acid was examined using the SignalP 4.0 Server (http://www.cbs.dtu.dk/services/SignalP) (accessed on 10 February 2022), and the cleavage sites were predicted using Neuro pred (http://stagbeetle.animal.uiuc.edu/cgi-bin/neuropred.py) (accessed on 10 February 2022). The three-dimensional (3D) structure of *SjILP* was generated using I-TASSER software [24,25] and edited using Swiss-Pdb viewer (v4.1.0) [26]. Multiple sequence alignments of ILP amino acid sequences were performed using Clustal W2 software (http://www.ebi.ac.uk/Tools/msa/clustalw2/) (accessed on 10 February 2022). The phylogenetic tree was constructed using the neighbor-joining (NJ) method (1000 bootstrap replicates) in MEGA5.0 [27]. The detailed information of these sequences is provided in Supplementary Table S1.

### 2.4. Subcellular Localization of SjILP

To investigate the subcellular localization of *Sj*ILP, HEK293 T cells were used as the in vitro model according to our previous reports [21,22]. HEK293 cells were cultured in Dulbecco’s modified Eagle’s medium (DMEM), supplemented with 10% FBS and 2% P/S, and maintained in a humidified incubator (37 °C with 5% CO_2_). The recombinant plasmids were constructed as described in a previous study. Cells were transfected with *Sj*ILP-pEGFP or pEGFP-N1 as a negative control using lipofectamine 2000 (Invitrogen) according to the manufacturer’s protocol. After transfection for 4 h, cells were fixed with 4% formaldehyde for 20 min and washed twice with PBS. Cytomembrane was stained with Dil for 20 min in the dark. Then, nuclei were stained with DAPI for 10 min at 25 °C. Subcellular localization of *Sj*ILP was observed using a digital confocal microscope (Leica TCSSP5, Germany). 

### 2.5. Expression of SjILP in Different Tissues

Total RNA was isolated from eight tissues, namely, the brain, liver, pancreas, muscle, heart, ovary, gill, and intestine. The RNA extraction and the cDNA synthesis process were performed as described in the present study. The expression of *Sj*ILP in different tissues was analyzed using quantitative Real-Time PCR (qRT-PCR). β-actin was applied as the internal reference gene [28]. Gene-specific primers and reference genes are listed in Table S4. The system and procedure of qRT-PCR were as described in our previous study [21]. Briefly, PCR reactions were performed in a medium with a volume of 10 μL, containing 5 μL TB Green™ Premix Ex Taq™ II, 0.4 μL of each primer (10 μM), 0.4 μL cDNA template, 0.2 μL ROX Reference Dye II, and 3.6 μL ultrapure H_2_O. The cycling protocol was as follows: initial incubation at 94 °C for 10 min, followed by 40 cycles of 95 °C for 15 s, 60 °C for 45 s, and 72 °C for 2 min. The relative gene expression was calculated using the 2^–ΔΔCT^ method [29]. Each sample was tested in three replicates.

### 2.6. Expression of SjILP in Different Ovarian Development Stages

To evaluate the expression of *Sj*ILP during the reproductive cycle of female *S. japonica*, tissues of the pancreas, ovary, and liver from four ovarian development stages were dissected. The RNA extraction, cDNA synthesis, and qRT-PCR process were performed as described above in the present study. Each sample was tested in three replicates.

### 2.7. RNA Interference In Vivo 

#### 2.7.1. Synthesis and Injection of dsRNA

Based on the complete sequence of *Sj*ILP, two dsRNAs and one negative control dsRNA were designed and synthesized using RT-PCR with primers containing the T7 polymerase promoter sequence (Table S4). The dsRNAs were dissolved in DEPC water to obtain a final concentration of 2 μmol/mL.

About 32 cuttlefish were randomly divided into four groups (*n* = 8 each group): two treatment groups (dsRNA1 and dsRNA2 injected) and two control groups (scramble dsRNA and normal saline injected). Then, 100 μL dsRNAs (dsRNA1 or dsRNA2) of *Sj*ILP, scramble dsRNA, or normal saline (NS) was injected according to the procedure described by Gore et al. [30]. At 48 h after injection, the ovary of each group was dissected. 

#### 2.7.2. Functional Analysis of SjILP with Gene Silencing

To evaluate the possible functions of *Sj*ILP in *S. japonica* reproduction, a highly effective dsRNA was selected to knockdown ILPs in females to examine their impact on the expression of ovarian-development-related genes. Four genes were selected, namely, vitellogenin (Vg1 and Vg2), cathepsin L1-like (CtsL1-like), and follistatin (FS). The expressions of these genes were detected using qRT-PCR as described above. Primers for these genes (Table S1) were designed based on sequencing information from the transcriptome data of *S. japonica* [19].

### 2.8. Statistic Analysis

Data are shown as mean ± standard deviations. All values were analyzed using one-way analysis of variance (ANOVA), and then followed by a multiple comparison in SPSS 14.0. The statistical significance was set at *p* < 0.05.

## 3. Results

### 3.1. Characterization of SjILP Sequence

**Figure 3:**
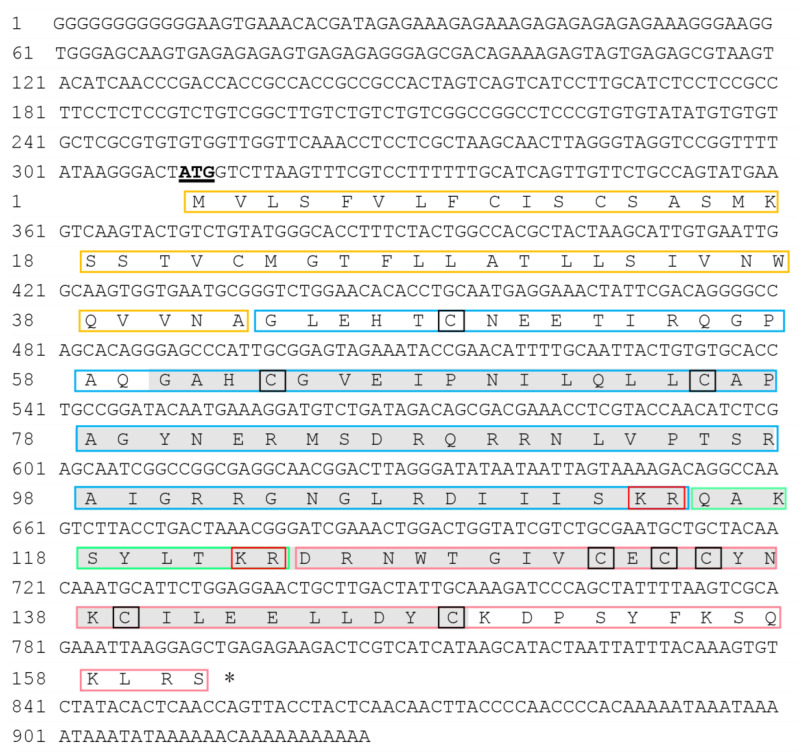
The nucleotide and deduced amino acid sequences of *S. japonica* ILP. The amino acid sequence of ORF consisted of a signal peptide (marked with yellow squares), B chain (marked with blue squares), C-peptide (marked with green squares), and A chain (marked with pink squares). Asterisk marks the stop codon. The putative cleavage sites and eight conserved cysteine residues are marked with red and black boxes, respectively. An insulin-like growth factor (IlGF) domain (aa 60–148) is indicated by a gray background.

**Figure 4:**
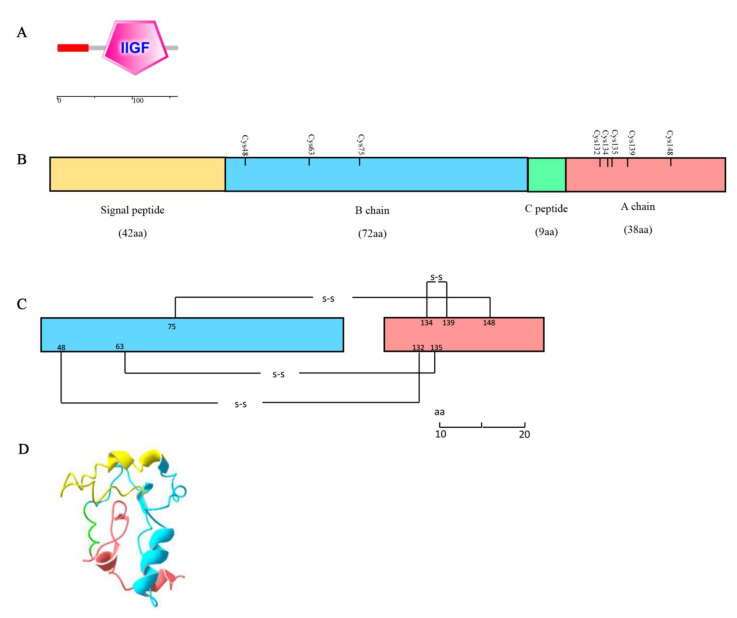
*Sj*ILP putative structure. (**A**) Domain architecture of prepro-*Sj*ILP. SMART-defined architecture of *Sj*ILP: a putative signal peptide followed by an insulin-like growth factor (IIGF) domain (aa 60–148). (**B**) Linear model of the 161 aa *Sj*ILP pre-prohormone, composed of a signal peptide of 42 aa (yellow), B chain of 72 aa (blue), C-peptide of 9 aa (green), and A chain of 38 aa (pink); eight cysteine residue positions are marked. (**C**) Linear model of mature *Sj*ILP. Disulfide bridges between C48-C132, C63-C135, C75-C148, and C134-C139 allow functional tertiary structure. (**D**) Suggested 3D model of *Sj*ILP, structurally based on Homo sapiens insulin.

The full-length ILP cDNA obtained from *S. japonica* was 926 bp. It contained a 310-bp 5′-untranslated region (UTR), a 486 bp open reading frame (ORF), and a 130 bp 3′-UTR (Figure 3). Bioinformatics analysis revealed that the ORF encoding a 161 amino acid (aa) polypeptide contained an insulin-like growth factor (IIGF) domain (aa 60–148, Figure 4A). Further analyses of this sequence identified two putative cleavage sites (arginine and lysine, KR) and eight conserved cysteine residues. The signal peptide and C-peptide of the ILP prepropeptide cleaved off to form a mature *ILP* comprising B and A chains (Figure 4C). These eight cysteine residues formed three inter-chain disulfide bonds (Cys48-Cys132, Cys63-Cys135, and Cys75-Cys148) and one intra-chain disulfide bond (Cys134-Cys139) (Figure 4C). In addition, its A chain presented a signature motif of MIPs: C(1X)CC(3X)C(8X)C. Based on these key features, the sequence obtained from *S. japonica* was termed *SjILP* (GenBank: MK611805).

Based on Homo sapiens insulin (PDB 7KD6) (sequence identity of 35.0%), the putative 3D structure of the mature protein was obtained, and it showed a similar structure with a TM score of 0.485 (Figure 4D). This similarity also demonstrates the conserved structure of *Sj*ILP at the tertiary level.

### 3.2. Multiple Alignment and Phylogenetic Analysis 

**Figure 5:**
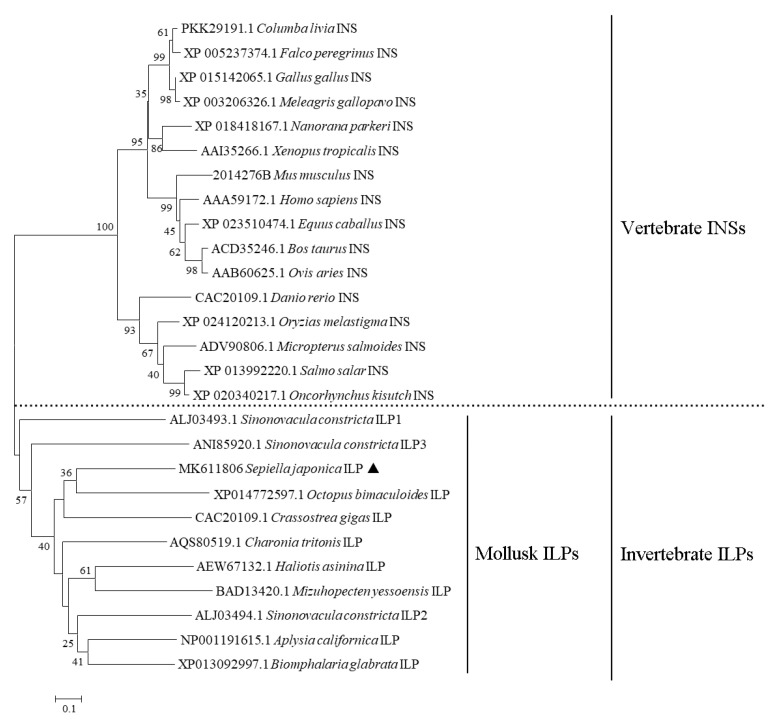
Phylogenetic tree based on amino acid sequences of mollusk ILPs and vertebrate INSs deposited in GeneBank. Neighbor-joining phylogram was constructed with 1000 bootstrap replicates in MEGA 6. *Sepiella japonica* ILP is indicated by a triangle.

The predicted mature peptide was further analyzed for sequence similarity with published sequences of *insulin*s and ILPs. The result revealed that their primary sequences were poorly conserved, except for cysteine residues (Figure S1). BLAST analysis revealed the amino acid sequence shared a high identity with the ILP for *Sepia latimanus* (98.14%). However, the identity between *S. japonica* and other mollusk orders was relatively low (Table S2). The sequence similarity results were further reflected in a phylogenetic tree constructed with the neighbor-joining method. As shown in Figure 5, *insulin* and ILPs were clustered together according to phylum and formed two clades: vertebrate *insulin*s and invertebrate ILPs. All molluskan ILPs formed a subgroup in which *Sj*ILP was most similar to the *Sepia latimanus* ILP. 

### 3.3. Subcellular Localization of SjILP

**Figure 6:**
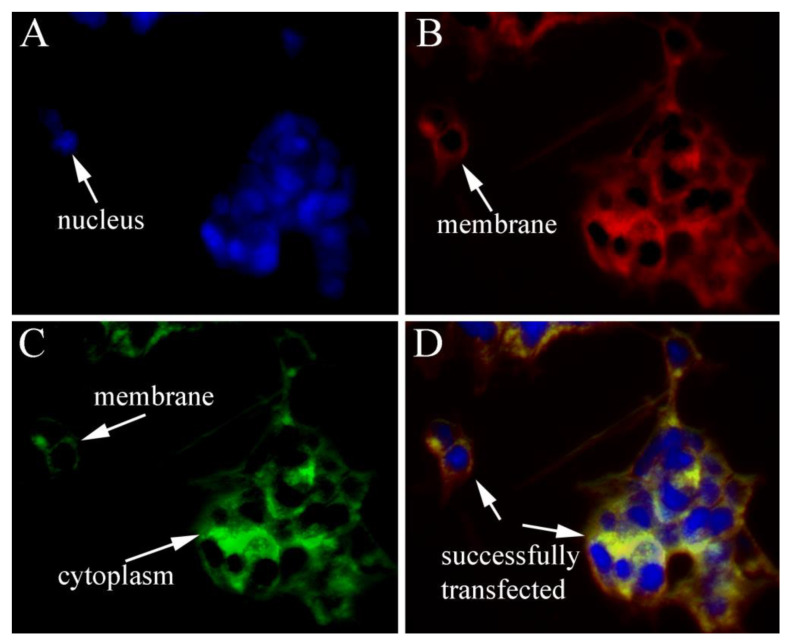
*Sj*ILP-EGFP fusion protein in HEK293 cells. (**A**) Nucleus of cells stained blue with DAPI. (**B**) Plasma membranes of cells stained red with DiI. (**C**) Cytoplasm and membrane of cells stained green with ERR-EGFP. (**D**) ILP was localized in the cytoplasm and the membrane of HEK293 cells; nucleus and the plasma membrane are indicated with white arrows in (**A**–**D**).

HEK293T cells were transfected with pEGFP-N1/*Sj*ILP to analyze the possible subcellular localization of *Sj*ILP. In contrast to the universal distribution of green fluorescence in the EGFP control, *Sj*ILP was localized in the cytoplasm and membrane, indicating the typical features of the secretory protein of *Sj*ILP (Figure 6).

### 3.4. Expression Profile of SjILP Transcript in Different Tissues 

**Figure 7:**
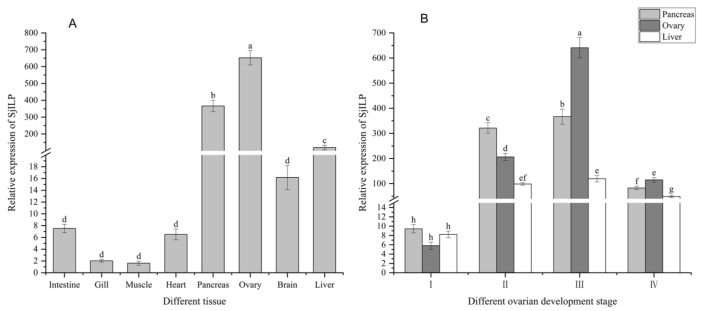
The expression of *Sj*ILP in different tissues (**A**) and ovarian development stages (**B**). Data are shown as means ± SD. Significant difference in *Sj*ILP mRNA levels is represented by different lowercase letters (*p* < 0.05).

As shown in Figure 7A, *Sj*ILP was expressed in all investigated tissues. However, the highest expression level was detected in the ovary, followed by the pancreas, liver, and brain. Moreover, its expression in the intestine, gill, muscle, and heart was relatively low (*p* < 0.05). Based on the tissue-specific expression profiles, the ovary, pancreas, and liver were used in expression detection for *Sj*ILP mRNA at different ovarian developmental stages.

### 3.5. Expression Profile of SjILP Transcript at Different Ovarian Developmental Stages

As shown in Figure 7B, low expression levels were detected at stage I, the expression level started to increase from stage II to stage III, the expression level reached the maximum at stage III, and then it obviously declined at stage IV.

### 3.6. Effects of SjILP Silencing on Ovarian Development 

**Figure 8:**
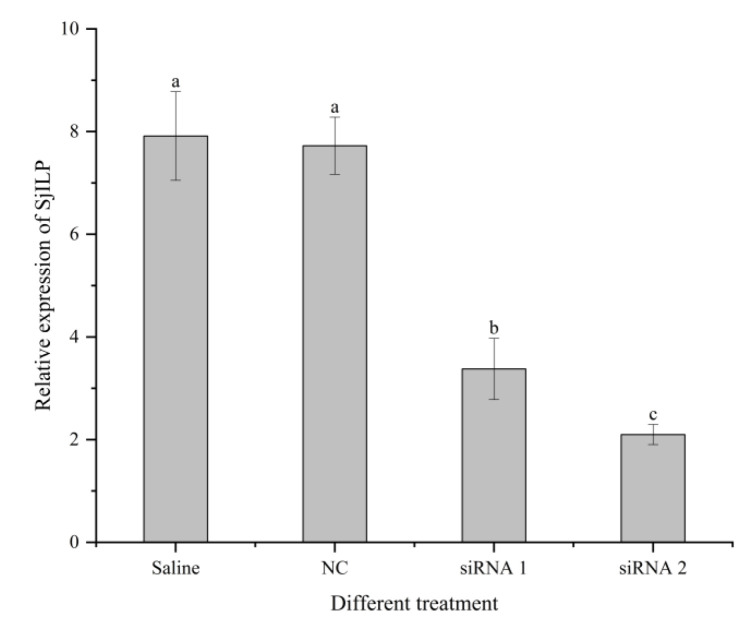
Effect of *Sj*ILP knockdown on the expression of *Sj*ILP. Data are expressed as mean ± SD (*n* = 8). Significant difference among different groups is indicated by the different lowercase letters (*p* < 0.05).

As shown in Figure 8, *Sj*ILP expression was effectively reduced by *Sj*ILP knockdown, with siRNA2 having the greater inhibitory effect (*p* < 0.05). Therefore, the dsRNA2-injected group was used as the treatment group to detect the expression of ovarian-development-related genes. 

**Figure 9:**
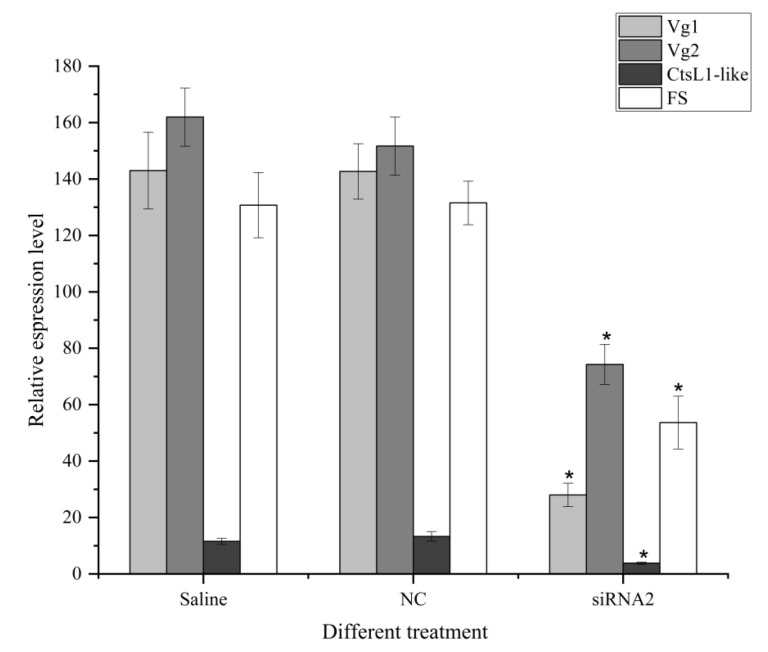
Effect of *Sj*ILP knockdown on the expression of reproduction-related genes. Vg1: vitellogenin1, Vg2: vitellogenin2, CtsL1-like: cathepsin L1-like, and FS: follistatin. Data are expressed as mean ± SD (*n* = 8). Significant differences (*p* < 0.05) are indicated by an asterisk (*).

As expected, the expression levels of Vg1, Vg2, CtsL1-like, and FS in the ovary were also significantly reduced by RNAi treatment (Figure 9), further indicating the role of *Sj*ILP in the ovarian development of *S. japonica*.

## 4. Discussion

In the present study, a putative ILP gene (*Sj*ILP) was identified from the cuttlefish *S. japonica*, and it shared a high similarity in gene organization with other molluskan ILPs. *Sj*ILP had two proteolytic cleavage sites (KR), which appeared to be highly conserved in lepidopteran ILP precursors (Figure 3) [31]. The C-peptide is removed from these two dibasic sites to form the mature ILP in the same way as vertebrate insulin [32]. Proteolytic cleavage of the C-peptide is a key post-translational modification for attaining hormone activity [33], which is a typical feature of the insulin family [34]. Combined with the finding that *Sj*ILP was localized in the cytoplasm and the membrane (Figure 6), these results suggest that *Sj*ILP belongs to the secretory protein. As is common to other molluskan ILPs, *Sj*ILP possessed eight cysteine residues constructing four disulfide bonds, which are essential for tertiary folding (Figure 4B,C) [5,6,35]. These cysteine residues also contribute to the hydrophobic core of insulins [4,35]. 

Although the structural organization of *Sj*ILP was similar to that of molluskan ILPs, it showed a low sequence identity (Table S1), possibly indicating the evolutionary divergence of ILPs in mollusks. This result is consistent with most previous reports about invertebrate ILPs [4,36]. Interestingly, in the various MIPs, not all the amino acid residues that are important for the maintenance of the basic conformation (i.e., the two-chain organization, proteolytic cleavage sites, and cysteine residues) are well-conserved; they are, in fact, only substitutions of surface residues that account for the differences between these peptides [37]. 

Gene expression is generally related to its function. In order to investigate the function of *Sj*ILP, we analyzed its expression in various tissues. In this study, it was found to be expressed in all tissues examined, which indicates a wide distribution and multiple functions of *Sj*ILP. The highest expression level of *Sj*ILP was found in the ovary, followed by the pancreas and liver (Figure 7A). This particular expression pattern of *SjILP* most likely reflects the specific demands of each tissue. The highest expression level in the ovary is in line with that reported by Iwami et al. [38], who found that the bombyxin transcript was localized in the *B. mori* ovary. Along with the report that bombyxin induced meiosis in *Bombyx* ovarian cells in vitro [39], our results suggest that *Sj*ILP plays an important role in ovarian development. Analogous findings have been reported for *C. gigas* [5]. In most vertebrates, insulin is primarily synthesized in endocrine pancreatic β cells, and it is mainly involved in growth control and carbohydrate metabolism [40]. The involvement of ILPs in the regulation of carbohydrate metabolism has also been reported in invertebrates, such as *Lymnaea stagnalis* [41] and *Aplysia californica* [3]. In this study, the relatively greater abundance of *Sj*ILP mRNA transcript in the pancreas suggests the paracrine function of insulin-like peptides during digestion (Figure 7A) [16]. In mollusks, neurosecretory cells in the cerebral ganglia are the main source of ILPs [42,43,44]. Moreover, in these animals, ILPs are not supposed to act as neurotransmitters on target neurons in the neural ganglia [37]. In this study, *Sj*ILP was also found to be expressed in the brain of *S. japonica* (Figure 7A), which may indicate a possible relationship between ILPs and neuroendocrine functional control in the brain to regulate reproduction. Other tissues, such as the intestine, heart, muscle, and gill, were also potential targets of insulin-like signaling, and they might be subjected to constant growth or cellular renewal [45].

Our analysis of *Sj*ILP expression during different ovarian development stages indicated that *SjILP* may regulate the reproduction of *S. japonica* (Figure 7B). The fluctuation of the *Sj*ILP expression level correlated with ovarian development, which suggests that ILP might be involved in ovarian development by stimulating oogenesis and/or vitellogenesis [46]. Combined with the finding that the insulin receptor (CIR) is expressed in the gonadal area of oysters and showed differential expression during embryogenesis and larval developmental stages [13,47], these results suggest that ILP might regulate reproduction through binding to its receptor. 

To further investigate the function of *Sj*ILP in the reproduction of *S. japonica*, we detected the expressions of ovarian-development-related genes (Vg1, Vg2, CtsL1-like, and FS) after knockdown of the *Sj*ILP gene in vivo. In squid, vitellogenin is the major precursor of egg yolk protein expressed predominantly in the ovary during ovary vitellogenesis/maturation [48]. Studies in insects, nematodes, and fish have indicated that ILPs positively regulate vitellogenesis [49,50,51]. Our data show that silencing the *Sj*ILP gene resulted in a decrease in Vg1 and Vg2 expression levels (Figure 9), which is in line with previous observations in *Schistocerca gregaria* [52]. These findings indicate that *Sj*ILP may indirectly control reproduction by affecting vitellogenesis. CtsL1 is an important member of the cysteine protease family and plays a crucial role in oocyte maturation [53]. In shrimp, Mn-CTS L1 has been verified to be involved in hydrolyzing vitellogenin before ovarian maturity to provide nutrients for the maturation of oocytes [54]. Furthermore, silencing cathepsin L using RNAi led to the accumulation of vitellogenin [55]. Here, the expression of CtsL1 mRNA markedly reduced after *Sj*ILP silencing, which suggests that *Sj*ILP possibly involves vitellogenesis in the control of reproduction in *S. japonica*. FS is a monomeric glycosylated protein that plays a key role in regulating folliculogenesis and ovarian development [56]. Similar to CtsL1, *Sj*ILP gene silencing also caused the down-regulation of FS in *S. japonica* (Figure 9). Although a direct influence of ILP on FS expression has not been proven in other species, the down-regulation of FS expression in *Sj*ILP-dsRNA-injected females suggests that *Sj*ILP possibly regulates reproduction by affecting the expression of ovarian-development-related genes in *S. japonica*. These findings show that an exogenous injection of *Sj*ILP dsRNA can effectively inhibit ovarian development, thereby providing a novel approach to reduce the rapid sexual maturation of female cuttlefish.

## 5. Conclusions

In summary, this study characterized the *Sj*ILP gene from *S.* *japonica*, and it further investigated the ovarian development mechanism. The highest expression level of *Sj*ILP mRNA was found in the ovary, and its gene expression correlated with ovarian development. In addition, the silencing of *Sj*ILP markedly down-regulated the expression of the ovarian-development-related genes. Our data add valuable information in investigating the molecular mechanism of ovarian development in cephalopods. However, to understand the regulative mechanism of *Sj*ILP in cephalopod reproduction, more detailed studies on the network of *Sj*ILP signaling will be necessary.

## Data Availability

The data that support the findings of this study are available from the corresponding author upon reasonable request.

## Supplementary Materials

The following supporting information can be downloaded at: https://www.mdpi.com/article/10.3390/cimb44060170/s1.
