# Supplementary material for: Characterization of Insulin-like Peptide (ILP) and Its Potential Role in Ovarian Development of the Cuttlefish Sepiella japonica
Source: Curr Issues Mol Biol. 2022 May 27;44(6):2490–504. doi: 10.3390/cimb44060170 (PMC9221753; doi:10.3390/cimb44060170)
Supplement: Supplementary file 1 [file cimb-44-00170-s001.zip › TableS1.pdf]

Table S1: Nucleotide sequences of primers and dsRNAs used

	Sequence (5'-3')	Usage
ILP-F	TGAGTAGGTAACTGGTTGAG	cDNA fragment cloning
ILP-R	AATTGGCAAGTGGTGAATG	
ILP 3'-F	CTGGAACACACCTGCAATG	3'-RACE
ILP 3'-R	ACAGGCCAAGTCTTACCTG	
ILP 5'-F	AGGTGCACACAGTAATTGC	5'-RACE
ILP 5'-R	TACTGGCAGAACAACTGATG	
RT-ILP-F	AACCTCGTACCAACATCTC	Real-time PCR
RT-ILP-R	CAATAGTCAAGCAGTTCCTC	
RT-Vg1-F	CACCTGCGACTGAACCTAAA	qPCR for Vg1
RT-Vg1-R	CAAGACGCTCAAGCAACATG	
RT-Vg2-F	GAGTCAGGCTTGCTATGG	qCR for Vg2
RT-Vg2-R	GTGGATTCATTACGGTCTAAG	
RT-CtsL1-like-F	GTATCTACTTACATACGCTGAC	qPCR for CtsL1-like
RT-CtsL1-like-R	AGTCCGAATAATGAGATGCT	
RT- FST-F	CTTGTCGTATGCGTGGAA	qPCR for FST
RT- FST-R	ATATAGGCGATACTGACTGAC	
RT-β-actin F	GCCAGTTGCTCGTTACAG	qPCR for β-actin
RT-β-actin R	GCCAACAATAGATGGGAAT	
negative control- sense	UUCUUCGAACGUGUCACGUTT	RNA interference
negative control- antisense	ACGUGACACGUUCGGAGAATT	
*Sj*ILP siRNA1- sense	GCCAGUAUGAAGUCAAGUATT	RNA interference
*Sj*ILP siRNA1- antisense	UACUUGACUUCAUACUGGCTT	
*Sj*ILP siRNA2- sense	GCGAAUGCUGCUACAACAATT	RNA interference
*Sj*ILP siRNA2- antisense	UUGUUGUAGCAGCAUUCGCTT	
β-actin-F	GCCAGTTGCTCGTTACAG	Real-time PCR
β-actin-R	GCCAACAATAGATGGGAAT	
